# A Badge or Distinction Costume for Physicians

**Published:** 1888-01

**Authors:** 


### A BADGE OR DISTINCTIVE COSTUME FOR PHYSICIANS.

A suggestion (*in the Medical World*) by Dr. I. H. Stearns, of Michigan, that physicians be distinguished by a badge, or some peculiar costume, is being favorably noticed by certain medical journals. We don't know so well about that. If all members of the profession were alike honorable, it would be well enough. But the quacks and the more unworthy members of the profession would be the first to avail themselves of a distinction which, however honorable at first, would soon be brought into such disrepute that no high-toned or worthy member of the profession would adopt it. We well remember the time when every preacher of the Methodist church wore a shad-bellied coat as a distinctive mark of that denominatlon. Bad men sometimes donned this peculiar costume and imposed themselves upon the public. It was related of old Judge Kennan, a prominent and somewhat eccentric man amongst the early settlers of upper Georgia, that on one occasion a traveller with a shad-bellied coat asked to spend the night with him. He bluntly refused the man, who started off cursing and swearing, whereupon the Judge called him back, and apologized, remarking: "I thought you were one of them d——d Methodist preachers."

